# Late-Onset Group B Streptococcal Septic Arthritis of the Shoulder and Proximal Humeral Osteomyelitis Presenting With Reduced Spontaneous Arm Movement in an Afebrile Infant

**DOI:** 10.7759/cureus.116981

**Published:** 2026-09-26

**Authors:** Ayaka Yamaoka, Soichiro Ishimaru, Taichi Kobayashi, Kentaro Suzuki, Shun Imamura

**Affiliations:** 1 Department of Pediatrics, Kariya Toyota General Hospital, Kariya, JPN

**Keywords:** group b streptococcus (gbs), late-onset gbs disease, osteomyelitis, reduced spontaneous movement, shoulder septic arthritis

## Abstract

We report a case of a one-month-old girl with late-onset group B *Streptococcus* (GBS) septic arthritis of the shoulder and proximal humeral osteomyelitis who presented with persistent reduction in spontaneous movement of the right upper extremity without fever, swelling, erythema, or warmth. Initial radiography showed no fracture, dislocation, or bone destruction. Because the reduced arm movement persisted, magnetic resonance imaging (MRI) performed approximately three weeks after symptom onset revealed findings suggestive of right shoulder septic arthritis and proximal humeral osteomyelitis. Blood and joint fluid cultures yielded *Streptococcus agalactiae*, identified as serotype III, confirming GBS bacteremia and septic arthritis, whereas proximal humeral osteomyelitis was diagnosed based on MRI findings. She underwent surgical irrigation and debridement followed by six weeks of antimicrobial therapy. Her right upper-limb function gradually improved, and no functional asymmetry was observed at two years and one month of age, although delayed development of the proximal humeral epiphyseal ossification center had been noted. This case emphasizes that late-onset GBS osteoarticular infection should be considered in young infants when unilateral reduction in limb movement persists for several weeks, even in the absence of fever or overt local inflammatory signs.

## Introduction

Group B *Streptococcus *(GBS) is a leading cause of invasive infection in neonates and young infants, and bacteremia and meningitis are the most common manifestations of late-onset GBS disease [1]. Osteoarticular infections, including osteomyelitis and septic arthritis, are uncommon manifestations of late-onset GBS disease overall; however, GBS remains an important pathogen in osteoarticular infections among infants younger than three months [2,3]. In young infants, osteoarticular infection may occur without fever or overt local inflammatory findings, and reduced spontaneous movement of the affected limb may be mistaken for brachial plexus palsy or birth trauma [4].

When reduced limb movement occurs in isolation and persists for several weeks, the absence of fever or overt local inflammatory signs may contribute to delayed recognition of osteoarticular infection. Delayed recognition is clinically important because bone and joint infections in early infancy may result in subsequent growth-plate injury, limb-length discrepancy, deformity, or functional impairment [5,6]. We report a case of late-onset GBS septic arthritis of the shoulder with proximal humeral osteomyelitis in an afebrile infant whose predominant clinical manifestation was a three-week history of persistent reduction in spontaneous movement of the right upper extremity without overt local inflammatory signs.

## Case presentation

A one-month-old girl was born at 39 weeks and 5 days of gestation with a birth weight of 3,094 g. She was delivered vaginally without shoulder dystocia or other birth trauma. Her Apgar scores were 8 and 9 at 1 and 5 minutes, respectively, and her neonatal course was unremarkable. She had no relevant medical history or known allergies.

Four days before her routine one-month health examination, her family noticed reduced spontaneous movement of her right upper extremity. At the examination, she was afebrile, feeding well, and gaining weight appropriately. The asymmetry was confirmed on examination, whereas finger movement and grasping were preserved. No erythema, swelling, or warmth was evident over the right shoulder. Plain radiographs showed no fracture, dislocation, or bone destruction, and she was initially followed up as an outpatient (Figure 1A).

**Figure 1:**
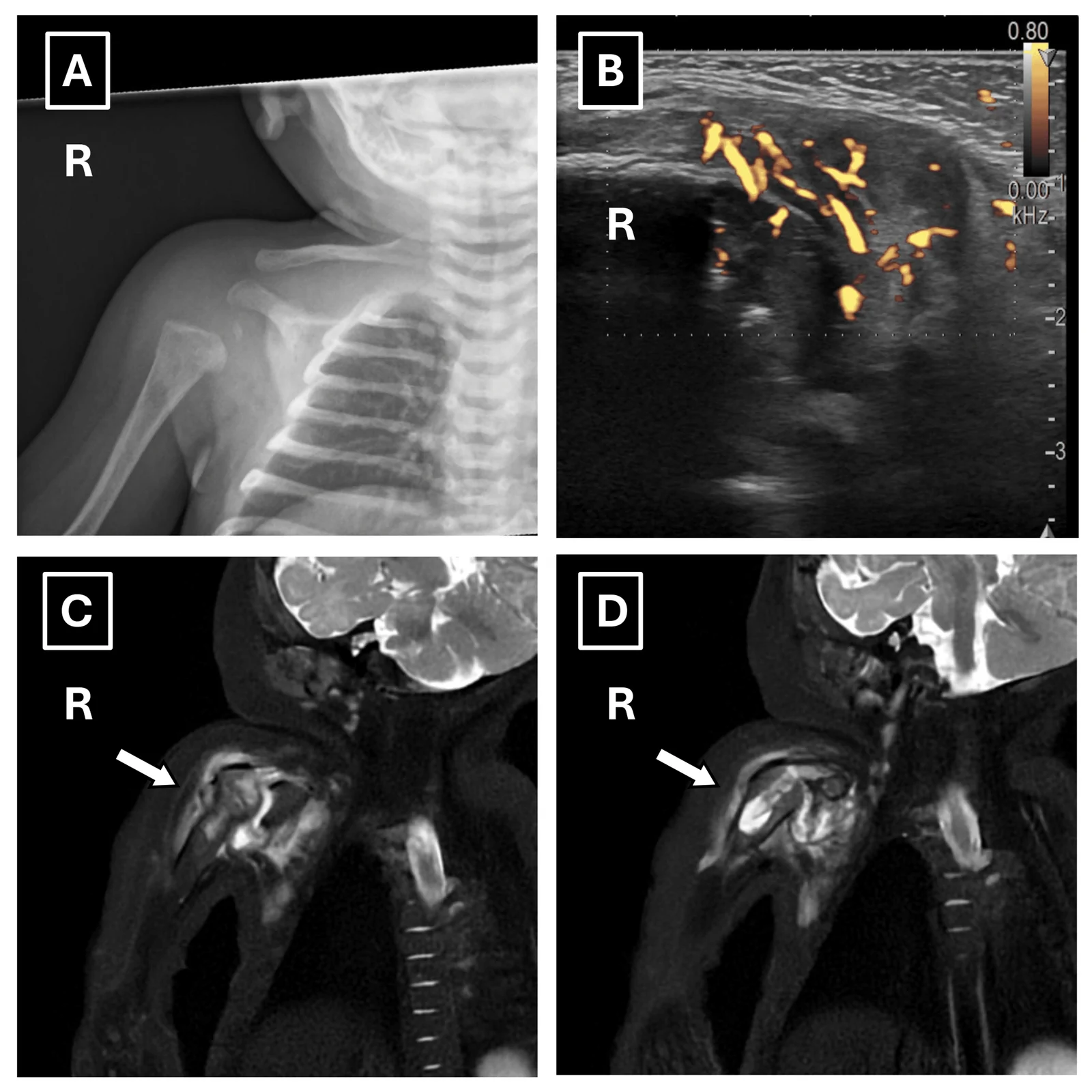
Imaging findings at initial evaluation and diagnosis (A) Initial plain radiograph showing no fracture, dislocation, or bone destruction. (B) Longitudinal power Doppler ultrasonographic image of the right shoulder obtained at diagnosis, showing increased periarticular vascularity. (C, D) Coronal fat-suppressed T2-weighted magnetic resonance images obtained at diagnosis, showing right shoulder joint effusion, periarticular inflammatory changes, and abnormal high signal intensity in the proximal right humerus (arrows).

Because the asymmetry persisted, MRI was performed 17 days after the initial assessment, approximately three weeks after symptom onset. MRI abnormalities around the right shoulder prompted admission for further evaluation and treatment.

On admission, her temperature was 37.4°C, and her vital signs were stable. Although tenderness of the right shoulder could not be clearly elicited, she cried intensely when the right upper extremity was elevated, and spontaneous movement at the shoulder was markedly reduced. Laboratory evaluation showed a white blood cell count of 15,900/μL, C-reactive protein level of 5.43 mg/dL, platelet count of 753 × 10³/μL, and procalcitonin level of 0.1 ng/mL.

Ultrasonography demonstrated synovial thickening, periarticular fluid collection, and increased vascularity on power Doppler imaging (Figure 1B). MRI revealed right shoulder joint effusion, inflammatory changes in the surrounding soft tissues, and high signal intensity in the proximal right humerus on fat-suppressed T2-weighted images (Figures 1C, 1D). These findings suggested right shoulder septic arthritis and proximal humeral osteomyelitis.

She underwent surgical irrigation and debridement of the right shoulder on the day of admission. Blood and joint fluid cultures grew *Streptococcus agalactiae*, which was identified as serotype III. Intravenous ampicillin (200 mg/kg/day) and cefotaxime (150 mg/kg/day) were initiated. Cefotaxime was discontinued on hospital day 5 after culture and antimicrobial susceptibility results became available, and ampicillin monotherapy was continued. The drain was removed on postoperative day 2. Blood and joint fluid cultures confirmed GBS bacteremia and septic arthritis, whereas proximal humeral osteomyelitis was diagnosed based on the MRI findings.

Antigravity movement of the right upper extremity was observed by hospital day 3 and improved further by hospital day 5. Pain during passive movement subsequently resolved, although spontaneous shoulder movement remained reduced for approximately three weeks. MRI on treatment day 27 and serial ultrasonography showed marked improvement in the joint effusion and surrounding inflammatory changes. She was transitioned from intravenous ampicillin to oral amoxicillin before discharge and completed a total of six weeks of antimicrobial therapy at three months of age. Cultures of breast milk and maternal urine and stool were negative, and the source of infection remained unidentified. The acute diagnostic and therapeutic course is summarized in Figure 2.

**Figure 2:**
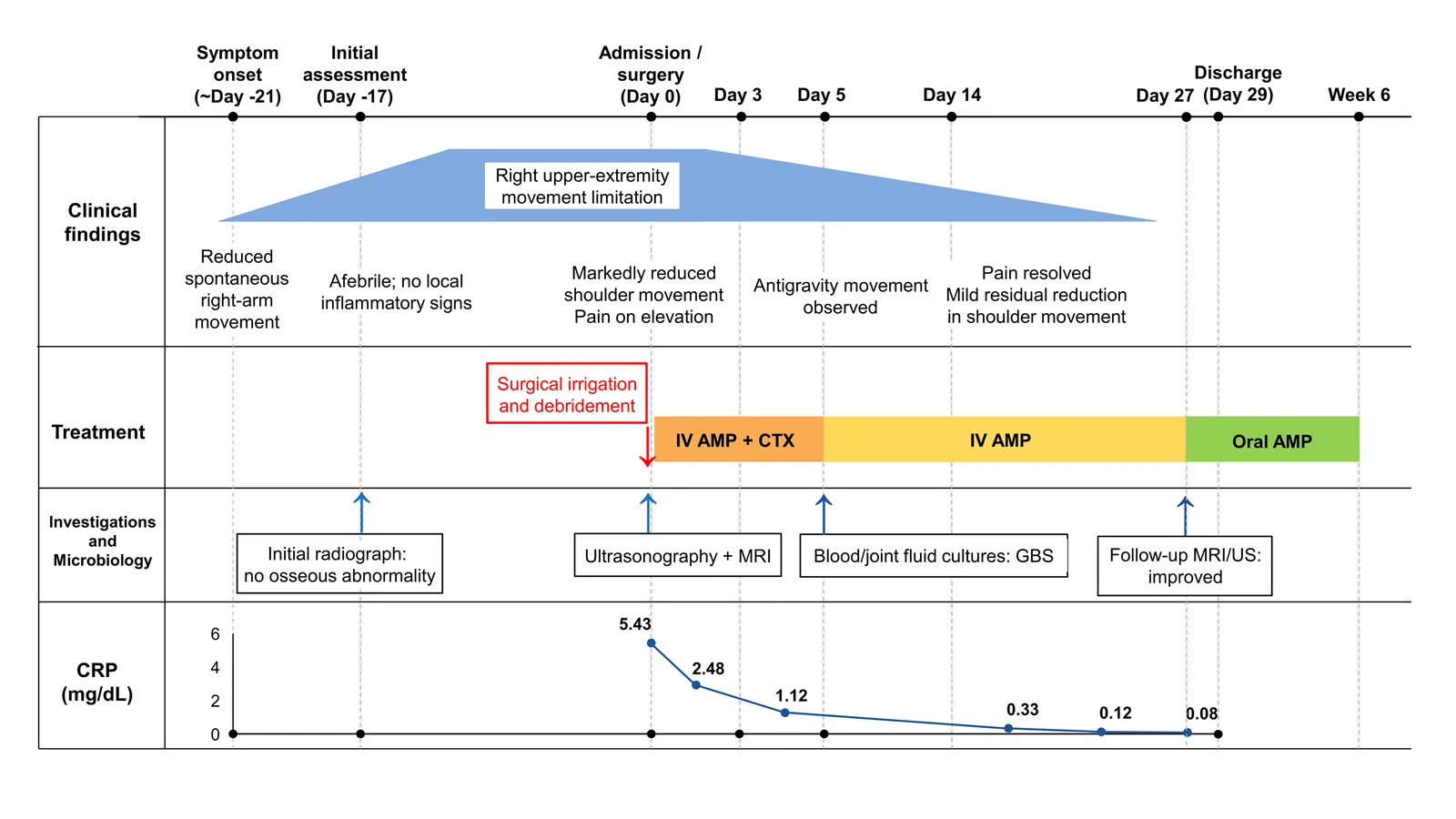
Clinical course from symptom onset to completion of antimicrobial therapy AMP, ampicillin; AMX, amoxicillin; CTX, cefotaxime; CRP, C-reactive protein; GBS, group B *Streptococcus*; MRI, magnetic resonance imaging; US, ultrasonography Reduced spontaneous movement of the right upper extremity persisted despite the absence of fever or local inflammatory signs at the initial assessment, and a diagnosis was established approximately three weeks after symptom onset. Upper-extremity movement and C-reactive protein levels improved after surgical irrigation and debridement and antimicrobial therapy. The shaded area schematically represents the course of right upper-extremity movement limitation. The figure was created by the authors using Microsoft PowerPoint (Microsoft Corporation, Redmond, USA).

Follow-up radiographs obtained approximately six months after diagnosis showed underdevelopment of the right proximal humeral epiphyseal ossification center compared with the contralateral side. The ossification center gradually enlarged on serial radiographs, with interval development evident approximately two years after diagnosis. By one year of age, spontaneous movement of the right shoulder was comparable to that of the contralateral side. At two years and one month of age, no asymmetry in upper-extremity movement or clinically apparent upper-limb length discrepancy was evident. However, continued follow-up is warranted because later growth-related sequelae cannot yet be excluded.

## Discussion

Although osteoarticular infections are uncommon manifestations of late-onset GBS disease, GBS remains an important causative pathogen in infants younger than three months [1-3]. In the series reported by Branco et al., among 24 infants younger than three months with osteoarticular infections, a causative pathogen was identified in 21 cases, of which 5 (23.8%) were caused by GBS [3].

Osteoarticular infection in young infants may present as reduced use of the affected limb or pseudoparalysis, although local swelling and warmth are also commonly observed. In a multicenter study by Sun et al., localized swelling was reported in 88.7%-98.9% of neonates and infants with osteoarticular infections, reduced spontaneous movement in 79.8%-95.7%, and local warmth in 69.2%-85.1% [7]. In our patient, reduced spontaneous movement of the right upper extremity persisted for approximately three weeks without fever, swelling, erythema, or warmth before the diagnosis was established. The prolonged duration of this isolated and clinically subtle manifestation was the principal diagnostic challenge in this case.

Several previously reported cases of GBS osteoarticular infection in infants have also presented with reduced upper-extremity movement (Table 1) [4,8,9].

**Table 1:** Selected reported cases of group B streptococcal osteoarticular infection in infants GBS, group B *Streptococcus*; MRI, magnetic resonance imaging; NR, not reported Time to diagnosis was calculated from the onset of the presenting symptom, when reported.

Reference/case	Age/sex	Site	Main presentation	Fever and local signs	Time from symptom onset to diagnosis	Basis/notes
Present case	1 month, female	Right shoulder septic arthritis; proximal humeral osteomyelitis	Reduced right-arm movement; distal movement preserved	Afebrile; no swelling, erythema, or warmth	Approx. 21 days	Normal initial radiograph; MRI/US suggested infection; blood and joint fluid cultures yielded GBS serotype III
Sadleir and Connolly [4], Case 1	3 days, male	Left humeral osteomyelitis	Acute left-arm monoplegia; pain with movement	Afebrile; shoulder warm and swollen	12 days	Normal early radiograph; later lytic lesion; blood culture yielded GBS
Sadleir and Connolly [4], Case 2	15 days, male	Left proximal humeral osteomyelitis	Reduced left-arm movement; flaccid arm	Afebrile initially; fever 38.5°C after 10 h; local signs NR	Approx. 1 day	Normal radiograph and US; bone scan positive; repeat blood culture yielded GBS
Sadleir and Connolly [4], Case 3	21 days, female	Left proximal humeral osteomyelitis	Absent left-arm movement; pain with passive motion	Afebrile; mild shoulder swelling	Approx. 18 days	Repeat radiograph and bone scan positive; synovial fluid culture yielded GBS
Hasegawa et al. [8]	31 days, female	Left shoulder septic arthritis; suspected proximal humeral osteomyelitis	Reduced use of the left arm	Afebrile; no swelling or erythema	13–14 days	Imaging suggestive; surgery performed on day 14
Lee et al. [9]	5 weeks, NR	Right shoulder septic arthritis; proximal humeral osteomyelitis	Reduced right-arm movement	Afebrile; no swelling or erythema	5 days	MRI suggestive; operative specimens confirmed infection

Among these cases, the interval from symptom onset to diagnosis and the presence of fever or local inflammatory signs varied substantially. In the present patient, diagnosis was established approximately 21 days after symptom onset despite the continued absence of fever or overt local inflammatory findings. Sadleir and Connolly described one afebrile infant diagnosed after approximately 18 days, although mild shoulder swelling was present [4]. Hasegawa et al. reported a case of an afebrile infant presenting without swelling or erythema in whom diagnosis was established after 13-14 days [8], whereas the patient described by Lee et al. was diagnosed after five days [9]. These observations emphasize that persistent unilateral reduction in spontaneous arm movement should itself prompt consideration of osteoarticular infection, even when other clinical signs of infection are absent.

During infancy, transphyseal vessels connecting the metaphysis and epiphysis remain present, facilitating extension of infection across the physis into the epiphysis and adjacent joint [10]. The coexistence of proximal humeral osteomyelitis and shoulder septic arthritis in our patient is consistent with this anatomic characteristic.

Because the unilateral reduction in arm movement persisted, further evaluation ultimately included laboratory testing, ultrasonography, and MRI. MRI demonstrated findings suggestive of shoulder septic arthritis, proximal humeral osteomyelitis, and surrounding soft-tissue inflammation. In retrospect, persistence of the unilateral movement abnormality despite the absence of fever or overt local inflammatory signs could have prompted earlier laboratory evaluation and advanced imaging. Thus, the persistence of this symptom, rather than the result of any single initial imaging modality, should be recognized as an important trigger for further evaluation.

Early diagnosis is important not only for controlling infection but also for minimizing subsequent effects on bone growth and joint function. In our patient, surgery and antimicrobial therapy resulted in the resolution of the infection and recovery of right upper-extremity function. Although no asymmetry in upper-extremity movement or clinically apparent upper-limb length discrepancy was evident at two years and one month of age, underdevelopment of the right proximal humeral epiphyseal ossification center had been observed during follow-up. In neonates and young infants, physeal injury after osteoarticular infection may become apparent only later despite satisfactory functional recovery [5,6]. Therefore, later growth-related sequelae cannot yet be excluded in our patient, and continued follow-up through skeletal maturity is warranted, with particular attention to humeral length discrepancy, osseous deformity, and limitation of shoulder range of motion.

## Conclusions

Herein, we described a case of an afebrile infant with late-onset GBS septic arthritis of the shoulder and proximal humeral osteomyelitis who presented with persistent reduction in spontaneous movement of the right upper extremity without overt local inflammatory signs. In infants younger than three months, unilateral reduction in limb movement that persists for several weeks should prompt consideration of osteoarticular infection, including late-onset GBS disease, even when fever and local inflammatory signs are absent. Recognition of this persistent but clinically subtle manifestation may facilitate earlier laboratory evaluation and advanced imaging. Because later growth-related sequelae cannot yet be excluded, long-term follow-up through skeletal maturity is warranted.

## References

[1] Coggins SA, Puopolo KM (2024). Neonatal group B Streptococcus disease. Pediatr Rev.

[2] Memon IA, Jacobs NM, Yeh TF, Lilien LD (1979). Group B streptococcal osteomyelitis and septic arthritis. Its occurrence in infants less than 2 months old. Am J Dis Child.

[3] Branco J, Duarte M, Norte S (2022). Osteoarticular infections in infants under 3 months of age. Pediatr Int.

[4] Sadleir LG, Connolly MB (1998). Acquired brachial-plexus neuropathy in the neonate: a rare presentation of late-onset group-B streptococcal osteomyelitis. Dev Med Child Neurol.

[5] Peters W, Irving J, Letts M (1992). Long-term effects of neonatal bone and joint infection on adjacent growth plates. J Pediatr Orthop.

[6] Saisu T, Kawashima A, Kamegaya M, Mikasa M, Moriishi J, Moriya H (2007). Humeral shortening and inferior subluxation as sequelae of septic arthritis of the shoulder in neonates and infants. J Bone Joint Surg Am.

[7] Sun K, Zhang C, Mao Z (2024). Clinical characteristics of neonatal and infant osteomyelitis and septic arthritis: a multicenter retrospective study. J Pediatr (Rio J).

[8] Hasegawa A, Mihata T, Fujiwara K, Noguchi Y, Neo M (2020). Arthroscopic treatment for septic arthritis of the shoulder in a 1-month-old infant: a case report. J Shoulder Elbow Surg.

[9] Lee B, Brown AG, Patel AM, Torok KS, Muhammad NA (2013). Index of suspicion. Pediatr Rev.

[10] Thakolkaran N, Shetty AK (2019). Acute hematogenous osteomyelitis in children. Ochsner J.

